# Conversion of Reactive Astrocytes to Induced Neurons Enhances Neuronal Repair and Functional Recovery After Ischemic Stroke

**DOI:** 10.3389/fnagi.2021.612856

**Published:** 2021-03-26

**Authors:** Michael Qize Jiang, Shan Ping Yu, Zheng Zachory Wei, Weiwei Zhong, Wenyuan Cao, Xiaohuan Gu, Anika Wu, Myles Randolph McCrary, Ken Berglund, Ling Wei

**Affiliations:** ^1^Department of Anesthesiology, Emory University School of Medicine, Atlanta, GA, United States; ^2^Center for Visual and Neurocognitive Rehabilitation, Atlanta Veterans Affair Medical Center, Decatur, GA, United States; ^3^Department of Neurosurgery, Emory University School of Medicine, Atlanta, GA, United States; ^4^Department of Neurology, Emory University School of Medicine, Atlanta, GA, United States

**Keywords:** ischemic stroke, direct reprogramming, induced neuron, glial scar, functional recovery, post-stroke depression

## Abstract

The master neuronal transcription factor NeuroD1 can directly reprogram astrocytes into induced neurons (iNeurons) after stroke. Using viral vectors to drive ectopic ND1 expression in gliotic astrocytes after brain injury presents an autologous form of cell therapy for neurodegenerative disease. Cultured astrocytes transfected with ND1 exhibited reduced proliferation and adopted neuronal morphology within 2–3 weeks later, expressed neuronal/synaptic markers, and extended processes. Whole-cell recordings detected the firing of evoked action potentials in converted iNeurons. Focal ischemic stroke was induced in adult GFAP-Cre-Rosa-YFP mice that then received ND1 lentivirus injections into the peri-infarct region 7 days after stroke. Reprogrammed cells did not express stemness genes, while 2–6 weeks later converted cells were co-labeled with YFP (constitutively activated in astrocytes), mCherry (ND1 infection marker), and NeuN (mature neuronal marker). Approximately 66% of infected cells became NeuN-positive neurons. The majority (~80%) of converted cells expressed the vascular glutamate transporter (vGLUT) of glutamatergic neurons. ND1 treatment reduced astrogliosis, and some iNeurons located/survived inside of the savaged ischemic core. Western blotting detected higher levels of BDNF, FGF, and PSD-95 in ND1-treated mice. MultiElectrode Array (MEA) recordings in brain slices revealed that the ND1-induced reprogramming restored interrupted cortical circuits and synaptic plasticity. Furthermore, ND1 treatment significantly improved locomotor, sensorimotor, and psychological functions. Thus, conversion of endogenous astrocytes to neurons represents a plausible, on-site regenerative therapy for stroke.

## Introduction

Ischemic stroke is a devastating disease with limited therapies available (Liu et al., 2014; Ginsberg, 2016; Catanese et al., 2017). Stem cell transplantation has emerged as a promising regenerative therapy for stroke due to its potential for repairing damaged brain structures and improving functional recovery (Liu et al., 2014; Wei et al., 2017). However, cell transplantation therapies face multiple obstacles including the hosts’ immune systems, poor transplanted cell survival, inappropriate migration/homing and differentiation, and the lack of specificity or integration into endogenous brain networks (Liu et al., 2014; Wei et al., 2017). Some clinical trials have also reported inconsistent results in the efficacy of cell transplantation therapies (Hatakeyama et al., 2020).

In the adult brain, neurogenesis mainly exists in two regenerative niches: the subventricular zone (SVZ) and the sub-granular zone (SGZ; Ohab and Carmichael, 2008). Unfortunately, endogenously generated cells are insufficient for enacting meaningful repair of damaged brain structures (Gogel et al., 2011). On the other hand, glial cells such as astrocytes exhibit great proliferative capacity (Khakh and Sofroniew, 2015). Astroglia are normally responsible for maintaining physiological homeostasis and supporting surrounding tissues by providing structural, trophic, and metabolic support to neurons while also playing a role in modulating synaptic activity (Chen and Swanson, 2003; Gibbs et al., 2008). Resident astrocytes in the brain remain mitotic throughout the lifespan and undergo rapid gliosis in response to injury. This characteristic response provides a rich source of cells adjacent to the site of injury (Liu and Chopp, 2016). After an ischemic insult, astrogliosis initially confers neuroprotective effects by forming a barrier to limit toxic substances (Pekny and Nilsson, 2005). As the gliotic tissue persists in subacute and chronic stages, the formation of a glial scar in the peri-infarcted region acts as physical and chemical barriers that release inhibitory paracrine factors which limit neuro-regeneration in and around the ischemic region (Sofroniew and Vinters, 2010; Pekny and Pekna, 2016). Thus, the accumulation of reactive astrocytes and astrogliosis during later phases after stroke is viewed as a major obstacle for regenerative therapy involving either endogenous or transplanted cells.

Several master transcription factors such as Neurogenic differentiation 1 (NeuroD1 or ND1), Neurogenin2 (Ngn2), Olig2, and Ascl1 have been identified for their role in controlling cell fate (Heinrich et al., 2010; Li and Chen, 2016). These discoveries provide a breakthrough opportunity to utilize endogenous and autologous cell substrates for regenerative therapy. More recently, the idea of direct reprogramming of non-neuronal cells allows for the trans-differentiation of glial cells (astrocytes, microglia, and oligodendrocytes) into induced neurons (iNeurons) without passing through a stem cell stage (Vignoles et al., 2019; Chen et al., 2020). Theoretically, this is a more efficient way to obtain desirable endogenous neurons from a large cellular pool for “on-site” repair in the brain (Torper et al., 2013; Vignoles et al., 2019). Being post-mitotic cells, iNeurons are not tumorigenic, which offers another advantage over naïve and differentiating stem cells.

Based on the efficiency and efficacy of glial cell reprogramming, we and others experimented with several combinations of transcription factors and settled on the use of the single neural transcription factor NeuroD1 (Guo et al., 2014; Gangal et al., 2017; Jiang et al., 2019b; Chen et al., 2020; Liu et al., 2020). Targeting astrocytes for neuronal reprogramming with different viral vectors has been tested in several animal models of neurodegenerative diseases including ischemic stroke with varying success (Guo et al., 2014; Rivetti di Val Cervo et al., 2017; Yamashita et al., 2019; Chen et al., 2020; Yavarpour-Bali et al., 2020). The exploration of this approach in animal disease models is at an early stage. The efficacy of neuronal conversion and its contribution to neuronal circuitry repair, the mechanisms involved in the regenerative process, and the functional benefits of this therapy have not been well defined. Concurrently, the specificity, reliability, and clinical potential of this novel regenerative gene therapy have sparked much debate. Investigations spanning various routes of administration and in animal models across the lifespan have yielded inconsistent results (Gresita et al., 2019). Our investigation examined the viability of reprogramming of astrocytes *in vitro* and *in vivo*. Reprogramming therapy was tested in a focal ischemic stroke model of rats, induced by occlusion of distal branches of the middle cerebral artery (MCA) accompanied with partial reperfusion (Wei et al., 1995; Jiang et al., 2017). This focal ischemic stroke represents common clinical cases of relatively small infarction followed by spontaneous or thrombolysis-induced partial recirculation (Hakim et al., 1987; Jorgensen et al., 1994; Neumann-Haefelin et al., 2004). The well-defined structure-function relationship of the damaged sensorimotor cortex is suitable for specific functional assessments during the acute, subacute, and chronic stages of stroke. After a stroke, we transduced ND1 using a lentivirus vector rather than other viral serotypes such as an adeno-associated virus (AAV) to preserve finer control over the scope of infection to study the mechanics of reprogramming on local circuitry and to limit the therapy to only the injured tissue. Neuronal network repair and functional recovery were confirmed using comprehensive assessments and behavioral tests up to 4 months after stroke.

## Materials and Methods

### Animals

A total of 72 C57BL/6 adult mice (male, 26–28 g, *n* = 6–8 per experimental group) were used in this investigation. For *in vitro* direct reprogramming experiments, we dissected astrocytes from P1 C57 mouse pups. The near-pure astrocyte (>98%) cultures featured little contamination by other cell types (Choi et al., 1987). Astrocyte to neuron reprogramming using a GFAP promoter creates a closed-loop feedback system whereby initial translation of ectopic ND1 is high in hypertrophied reactive astrocytes but diminishes as the cell reprograms into neurons (Choudhury and Ding, 2016). For *in vivo* experiments, we crossed GFAP-Cre with Rosa-YFP mice to generate animals that exhibit constitutively active YFP expression in astrocytes regardless of cell phenotype changes; this property allowed tracking the cell fate of reprogrammed cells from start to finish. In these mice, Cre recombinase expression is controlled by a GFAP promoter to inverts a loxP-flanked YFP in astrocytes. The resulting YFP reporter is both astrocyte-specific and remains activated independently of cell fate (McLellan et al., 2017). When astrocytes are reprogrammed into induced neurons, they no longer express GFAP but continue to express the YFP promoter while preexisting neurons do not express YFP. The specificity of Cre recombinase under a human GFAP promoter in mice has been thoroughly characterized. Specificity defined as the proportion of co-labeled S100β and reporter positive cells over the total number of reporter positive cells in the mouse has been reported as high as 96.02% (Park et al., 2018).

The animal protocol (DAR 2003027) was approved by the Institutional Animal Care and Use Committee (IACUC) of Emory University School of Medicine. Animal procedures followed institutional guidelines that meet NIH standards of principles of laboratory animal care (NIH publication No. 86-23). Animals were randomly assigned to different experimental groups and data were analyzed under blinded conditions.

### NeuroD1 Lentiviral Production, Purification, and Titer Calculation

To express genes together with mCherry using a single plasmid, pEGIP was modified by replacing the GFP-IRES-Puro sequence with *BamHI*-*EcoRI*-IRES-mCherry-WPRE (EIMW). pEGIP was a gift from Linzhao Cheng (Addgene plasmid #26777; Zou et al., 2009). The mCherry tag was ligated to FUGW using two-step overlap PCR. A purified ND1 fragment was ligated into the FUGW plasmid. Verification of correct ligation and plasmid generation was confirmed using PCR and DNA sequence analysis. Plasmid production utilized Stbl3 bacteria and DNA was purified using Qiagen Miniprep and Maxiprep kits. Transduction efficacy was assayed compared to control GFP-FUGW in HEK 293FT cell cultures that were fixed and stained for both mCherry and ND1. To prevent interference from the mCherry reporter with ectopic ND1 translation, an internal ribosome entry site (IRES) preceding mCherry ensures that it is translated separately from ND1 (Martinez-Salas et al., 2001). The resulting ND1 and mCherry products are independent and able to be transported throughout the cell separately. While both proteins are translated in the cytoplasm, transcription factors such as ND1 contain nuclear localization signals while mCherry remains throughout the cytoplasm (Petersen et al., 2002).

Cloning experiments were performed in triplicates. A minimum of eight samples were required during cloning steps and three bacterial strains were sequenced to isolate a mutation-free strain. Of these, two contained point mutations, and one was mutation-free. All subsequent experiments used the mutation-free clone. In transduction experiments, three wells of HEK-293 cells were used to examine efficacy. DNA concentrations were assessed using Gen5 ultraviolet spectrophotometry by BioTek (Naldini et al., 1996). Plasmids were isolated using the following primers:

**Table:** 

Ngn2-BamHI-F	5′-GGG GGATCC ATGTTCGTCA AATCTGAGA-3′
Ngn2-KpnI-myc-EcoRI-R	5′-CCCGAATTCT CACAGATCCT CTTCAGAGAT GAGTTTCTGC TCGGTACCGA TACAGTCCCT GGCGAGGG-3′
ND1-BamHI-F	5′-GGTGCCTTGCTATTCTAAGACGC-3′
ND1-EcoRI-R	5′-GCAAAGCGTCTGAACGAAGGAG-3′

Note: F, Forward primer; R, Reverse primer.

### Generate ND1 Plasmid by Cloning ND1 Under GFAP Promoter With mCherry Tag Into the FUGW Plasmid

Lentivirus has been established as an effective method of transduction both *in vitro* and *in vivo* (Naldini et al., 1996; Blömer et al., 1997; Taoufik et al., 2007). Under a GFAP promoter, we packaged control GFP-FUGW and ND1-FUGW into a lentiviral vector. HEK 293FT cells were used as the substrate for lentiviral production (Tiscornia et al., 2006; Ansorge et al., 2009). We achieved a viral titer of at least 2.48 × 10^7^ which was suitable for use *in vitro* and *in vivo*. The virus was harvested from culture media two times resulting in 144 ml total. To reach a high titer for *in vivo* use, the virus was concentrated using two rounds of ultracentrifugation to concentrate first from 144 to 1.2 ml and then from 1.2 to 30 μl. Phage titer was calculated by infecting triplicate HEK 293FT cell cultures with 1:10 and 1:100 dilutions of concentrated virus and then fixed and stained for mCherry.

### Astrocyte Cultures and Lentivirus Infection

Astrocytes were dissected and cultured as described (Li et al., 2013). Briefly, cerebral cortex from postnatal day 1 (P1), and cells were cultured in a medium consisting of Dulbecco’s Modification of Eagle’s Medium (DMEM, Corning, Manassas, VA, USA), 15% FBS (Sigma), MEM non-essential amino acids (Life Technologies), 3.5 mM glucose (Sigma). One-week post-plating, cells were dissociated using trypsin-EDTA (Life Technologies) and passaged onto poly-D-lysine (Sigma) and laminin (Sigma) coated coverslips (80,000 cells per well for 24-well plates) in the same medium. A 4-day-long shaking procedure helped to remove microglia and neurons. By the end of this regimen, astrocyte activation and GFAP expression were induced uniformly across the culture by the addition of lipopolysaccharide (LPS). Neurons contained in initial cultures died within 10 days in culture. GFAP-ND1 expression is reliant on GFAP-positive astrocytes of the reactive state. We confirmed that 98.4% of the cells were positive for GFAP in our P10 astrocyte cultures.

Lentivirus was added into astrocyte cultures immediately after passage. One day post-transduction, the medium was completely replaced to minimize exposure to viral adjuvants into a medium consisting of DMEM/F-12 (Life Technologies), 3.5 mM glucose, penicillin/streptomycin (Life Technologies), B27 (Life Technologies), and 20 ng/ml brain-derived neurotrophic factor (BDNF, Sigma). After an initial media change, half-media changes occurred every 3–4 days subsequently.

### Electrophysiological Examination of ND1 Converted Cells *In vitro*

Astrocyte cultures from 14 to 42 days *in vitro* after ND1 transduction were examined using whole-cell patch-clamp recordings on converted cells identified by the morphological phenotype of axonal outgrowth with neurite extension. Recordings were performed using an EPC9 amplifier (HEKA; Elektronik) were performed at 21–23°C. The external solution contained (in mM): 135 NaCl, 5 KCl, 1 MgCl_2_, 2 CaCl_2_, 10 HEPES, and 10 Glucose at a pH of 7.4. Recording electrodes pulled from borosilicate glass pipettes (Sutter Instrument) had a tip resistance between 5 and 8 MO when filled with the internal solution (in mM): 140 KCl, 2 MgCl_2_, 1 CaCl_2_, 2 Na_2_ATP, 10 EGTA, and 10 HEPES at a pH of 7.2. Action potentials were recorded under the current-clamp mode using the Pulse software (HEKA, Elektronik).

### MicroElectrode Array (MEA) Recording in Brain Slices

A high-resolution MEA2100-system (MultiChannel Systems, Reutlingen, Germany) was used to record field excitatory postsynaptic potentials (fEPSP) in brain slices. The MEA chamber (60pMEA200/30iR-Ti, MultiChannel Systems GmbH, Reutlingen, Germany) is composed of a 6-mm high glass ring and an 8 × 8 Titanium nitride electrode grid (59 electrodes and 1 internal reference electrode) with an electrode diameter of 30 μm and spacing of 200 μm. The brain slice was transferred to the MEA chamber that was perfused with oxygenated aCSF at a rate of 6–8 ml/min and stabilized at 34°C for at least 10 mins before recording. For evoked fEPSPs, electric stimuli (±1.5 V, 10 ms) were applied every 30 s and responses were simultaneously monitored in 58 locations. Pair-pulse facilitation was recorded by two electric stimuli (±1.0 V, 10 ms) with different intervals (20, 40, 60, 80, 100, 200 ms). The slopes of fEPSPs were analyzed with Multi-Channel Analyzer V 2.6.0 (Multichannel Systems) and GraphPad Prism 6 (GraphPad Software, San Diego, CA, USA).

### Focal Ischemic Stroke in Mice

A focal cerebral ischemic stroke targeting the right sensorimotor cortex was induced as previously described (Choi et al., 2012; Li et al., 2013). Mice were anesthetized with 3% isoflurane and maintained using 1.5% isoflurane supplemented with regular air during surgery. Cortical ischemia was achieved by permanent occlusion of the distal branches of the right middle cerebral artery (MCA) supplying the sensorimotor cortex. The MCA occlusion was paired with 7-min ligation of both common carotid arteries (CCAs) to cause sufficient reduction of local cerebral blood flow (LCBF) in the sensorimotor region and followed by partial reperfusion. This relatively small stroke targets a well-defined brain structure, i.e., the sensorimotor cortex including the barrel cortex (Wei et al., 1995; Jiang et al., 2017). According to the epidemiological data from American Heart Association (AHA), small strokes are common and represent about 40% of all stroke cases (Roger et al., 2011). Importantly, partial reperfusion due to incomplete (spontaneous and post-thrombolytic) recanalization after an ischemic attack occurs in 30–70% of clinical cases at different times after the onset of ischemia (Hakim et al., 1987; Jorgensen et al., 1994; Barber et al., 1998; Neumann-Haefelin et al., 2004). Few animal models of ischemic stroke featuring partial reperfusion have been available. In this regard, the investigation on this stroke model possesses high face validity for translational research.

Body temperature was monitored during surgery and recovery period using a rectal probe and maintained at 37°C on a homoeothermic blanket in a ventilated incubator. Overall mortality resulting from ischemic stroke surgery was less than 2%. Before and after surgery the painkiller meloxicam was administered orally at a dosage of 5 mg/kg. Animals were housed with four to five mice per cage, with *ad libitum* access to food and water. At different time points after stroke, mice were sedated with overdose isoflurane and sacrificed by decapitation.

### Stereotaxic Injection

Stereotaxic injection of control and ND1 lentivirus was performed using a 10 μL Hamilton GASTIGHT™ syringe (Hamilton Company, NV). Injection locations included three areas around the infarcted region (Stereotaxic coordinates: AP −0.5, ML +3.2, DV +2.2) at a cortical depth of 0.5 mm and the sub-ventricular zone (SVZ; Stereotaxic coordinates: AP 0, ML +1.5, DV +2.5). Infected cells could be observed up to a distance of 3–4 mm from the injection site.

At time points of 24 and 48 h after the onset of MCAO, animals were sacrificed for assessment of brain infarct formation. 2,3,5-triphenyltetrazolium chloride (TTC; Sigma–Aldrich) staining was used to reveal damaged/dead brain tissue as previously described (Wang et al., 2014). Brains were removed and placed in a brain matrix then sliced into 1-mm coronal sections. Slices were incubated in 2% TTC solution at 37°C for 5 min, then stored in 10% buffered formalin for 24 h. Digital images of the caudal aspect of each slice were obtained by a flatbed scanner. Infarct, ipsilateral hemisphere, and contralateral hemisphere areas were measured using ImageJ software (NIH, Bethesda, MD, USA). The indirect method (subtraction of residual right hemisphere cortical volume from cortical volume of the intact left hemisphere) was used for infarct volume calculation. Infarct measurements were performed under double-blind conditions.

### Western Blotting Analysis

Western blot analysis was used to detect the expression of trophic factors brain-derived neurotrophic factor (BDNF), FGF10, PSD-95, tyrosine hydroxylase (TH), NFKB, and GFAP (*n* = 3–6). Brain cortical tissue was lysed in a lysis buffer containing 0.02 M Na_4_P_2_O_7_, 10 mM Tris-HCl (pH 7.4), 100 mM NaCl, 1 mM EDTA (pH 8.0), 1% Triton, 1 mM EGTA, 2 mM Na_3_VO_4_, and a protease inhibitor cocktail (Sigma–Aldrich). The supernatant was collected after centrifugation at 15,000 *g* for 10 min at 4°C. Protein concentration was determined with a bicinchoninic acid assay (Pierce Biotechnology, Rockford, IL, USA). Equivalent amounts of total protein, 15–20 μl were added per lane, were separated by molecular weight on an SDS-polyacrylamide gradient gel, and then transferred to a polyvinyl difluoride (PVDF) membrane. The blot was incubated in 5% bovine serum albumin (BSA) for at least 1 h and then reacted with primary antibodies at 4°C for overnight. The primary antibodies used in this investigation included: anti-BDNF antibody (1:2,000; Cell Signaling, Danvers, MA, USA), anti-FGF-10 antibody (1:500, Abcam, Cambridge, MA, USA), anti-PSD-95 (1:750, Abcam), anti-TH antibody (1:1,000; Cell Signaling, Danvers, MA, USA), anti-FGF-10 antibody (1:500, Abcam), anti-GFAP antibody (1:500, Abcam), rabbit anti-actin antibody (1:500, Abcam), rabbit anti-beta tubulin antibody (1:500, Abcam) and rabbit anti-cleaved caspase-3 (1:500; Cell Signaling). After washing with Tris-buffered saline with Tween (TBST), membranes were incubated with AP-conjugated or HRP-conjugated secondary antibodies (GE Healthcare, Piscataway, NJ, USA) for 1–2 h at room temperature. After final washing with TBST, the signals were detected with bromochloroidolylphosphate/nitroblue tetrazolium (BCIP/NBP) solution (Sigma–Aldrich) or film. Signal intensity was measured by ImageJ and normalized to the actin signal intensity.

### Immunocytochemistry and Immunohistochemical Staining

For immunocytochemistry (ICC), cell cultures were fixed using 10% formalin buffer, washed with −20°C precooled ethanol: acetic acid (2:1) solution for 10 min, and finally permeabilized with 0.2% Triton-X 100 solution for 5 min. All slides were washed three times with PBS (5 min each) after each step. Then, tissue sections were blocked with 1% fish gelatin (Sigma–Aldrich) in PBS for 1 h at room temperature, and subsequently incubated with the primary antibody: mouse anti-Tuj1 (1:500; Covance/Biolegend, CA, USA), rabbit synaptophysin (1:500; Ab32127, Abcam, Cambridge, MA, USA), rabbit anti-synapsin I (1:500; Millipore, Billerica, MA, USA), and rabbit anti-mCherry (1:400; Abcam) overnight at 4°C.

For immunohistochemistry (IHC), frozen brain tissues were sliced into 10 μm-thick coronal sections using a cryostat vibratome (Leica CM 1950; Leica Microsystems, Buffalo Grove, IL, USA). The sections were dehydrated on a slide warmer for 30 min, fixed with 10% formalin buffer, washed with −20°C precooled ethanol: acetic acid (2:1) solution for 10 min, and finally permeabilized with 0.2% Triton-X 100 solution for 5 min. All slides were washed three times with PBS (5 min each) after each step. Then, tissue sections were blocked with 1% fish gelatin (Sigma–Aldrich) in PBS for 1 h at room temperature, and subsequently incubated with the primary antibody: mouse anti-NeuN (1:400; Millipore, Billerica, MA, USA), rabbit anti-ND1 (1:400; Millipore, Billerica, MA, USA), chicken anti-GFAP (1:400; Abcam), mouse anti-iba-1 (1:400; Sigma–Aldrich), and rabbit anti-Beclin (1:5,000; Abcam, Cambridge, MA, USA), goat anti-collagen type IV (1:400; Millipore), rabbit anti-mCherry (1:400; Abcam), rabbit anti-vGluT1 (1:500; Abcam), rabbit anti-GAD67 (1:500; Abcam), rabbit anti-Iba1 (1:400; Abcam), rabbit anti-vGluT1 (1:500; Abcam), mouse anti-GFP (1:500; Sigma–Aldrich), Oct4 (1:250, Cell Signaling, Danvers, MA, USA) and Klf4 (1:500, Abcam) overnight at 4°C.

For both ICC and IHC, on the next day, the samples were washed three times with PBS for 5 min, then reacted with the secondary antibodies Alexa Fluor^®^488 goat anti-mouse or rabbit (1:300; Life Technologies, Grand Island, NY, USA) and Cy3-conjugated donkey anti-rabbit (1:300; Jackson ImmunoResearch Laboratories, West Grove, PA, USA) or Cy5-conjugated donkey anti-mouse or rabbit (1:400; Jackson ImmunoResearch Laboratories) for 80 min at room temperature. After three washes with PBS, nuclei were stained with Hoechst 33342 (1:20,000; Molecular Probes, Eugene, OR, USA) for 5 min as a counterstain; and then the brain sections were mounted, coverslipped, imaged, and photographed under a fluorescent microscope (BX51, Olympus, Japan) or a laser scanning confocal microscope (Carl Zeiss Microimaging, Inc., Thornwood, NY, USA).

For H&E, brains were sectioned with a vibratome and coronal sections (20 μm) of mouse brains were collected from the sensorimotor cortex (brains damaged during removal were not sectioned). Every 6th section and a total of 10 sections per brain were selected for H&E staining from the anterior start to the posterior end of the injury site.

### Astroglia Scar Measurement

Glial scar measurements were taken using ImageJ against GFAP immunofluorescence contiguous to the ischemic core. The thickness of the glial scar was measured using the average distance across hypertrophied astrocytes lining the ischemic core based on a method previously described (Cai et al., 2017). GFAP area and intensity were determined using the ImageJ analysis of the area of GFAP-positive fluorescence in the glial scar region respectively. Median GFAP intensity was measured using a 100-pixel width rectangle across six different regions of the glial scar to measure median GFAP intensity across the area.

### Cell Death Assessment

A terminal deoxynucleotidyl transferase dUTP nick end labeling (TUNEL) assay kit was used to examine cell death by detecting fragmented DNA in 10-μm-thick coronal fresh frozen sections as described previously (Lee et al., 2014). After fixation (10% buffered formalin for 10 min and then ethanol:acetic acid (2:1) solution for 5 min) and permeabilization (0.2% Triton X-100 solution), brain sections were incubated in equilibration buffer for 10 min. Recombinant terminal deoxynucleotidyl transferase (rTdT) and nucleotide mixture were then added on the slide at 37°C for 60 min in the dark. Reactions were terminated by 2× SSC solution for 15 min. Nuclei were counterstained with Hoechst 33342 (1:20,000; Molecular Probes) for 5 min. Cell counting was performed following the principles of design-based stereology (Lee et al., 2014). Systematic random sampling was used to ensure accurate and non-redundant cell counting. Eight brain sections per animal were collected at 90 μm distance between sections for non-overlapping multistage random sampling. For each animal, 6 “areas of interest” regions per slide were selected. Each field was scanned at 200× magnifications for cell counting. ImageJ (NIH) was used to analyze each picture. All experiments were performed in a blinded fashion, so the data collector and data analysis were performed without knowledge of experimental groups.

### Functional and Behavioral Tests

#### Rotarod Test

Rats were placed on an accelerating rotarod cylinder (Economex, Columbus In., Columbus, OH, USA) and the length of time the animals remained on the rotarod was measured. The speed was slowly increased from 4 to 40 rpm in 5 min. The animals were trained for 3 days before stroke surgery. The mean duration on the device was recorded with three measurements (Wittenberg et al., 2007; Spurlin and Nelson, 2017).

#### Corner Test

The corner test monitors unilateral whisker deficits. Mice were allowed to roam freely in a star-shaped arena with 30° angles. Upon entering a corner, the mouse would rear and turn toward the wall, which was sensed by intact whisker sensations. In our right sensorimotor cortex ischemic stroke model, mice lost sensation in their left whiskers. The percentage of right turns was measured before and after stroke (Calabresi et al., 2003; McCrary et al., 2019).

#### Forced Swimming Test

The forced swim test (FST) was carried out to measure depressive behaviors. Mice were dropped individually into a plexiglass cylinder (height: 30 cm, diameter: 22.5 cm) filled with water to a depth of 15 cm and maintained at 23–25°C. In this test, after the initial vigorous activity of 2 min, mice acquired an immobile posture which was characterized by motionless floating in the water and made only those movements necessary to keep the head above water. The duration of immobility was recorded in seconds during the last 4 min of the 6 min test. All mice received a 15-min training session under similar conditions 24 h before the formal test (Kronenberg et al., 2014; Frechou et al., 2019).

#### Sucrose Preference Test

The mice were given access to both water and a sucrose solution, and their preference for the sucrose solution was quantified. Briefly, mice were deprived of food and water for 20 h. One bottle of water and one containing 1% sucrose were simultaneously placed in the cages and were freely accessible to the mice for 3 h. The position of the two bottles (left or right side of the cage) was varied randomly from trial to trial. The volume of each liquid was measured before and after each trial, and sucrose preference was calculated according to the following equation: sucrose preference = (sucrose consumption)/(sucrose consumption + water consumption) × 100 (Heins et al., 2002; Grealish et al., 2016).

#### Tail Suspension Test (TST)

Mice were taped to a crossbar by their tails and their behavior was recorded for 10 min. In this test, after the initial vigorous activity of 2 min, mouse behavior was during an analysis period of 6 min. The duration of immobility was recorded in seconds during the time. In our laboratory, small movements that are confined to the front legs but without the involvement of the hind legs are not counted as mobility. Additionally, oscillations and pendulum-like swings that are due to the momentum gained during the earlier mobility bouts also are not counted as mobility. The same blinded observer was used to assess mobility in all experiments (Ge et al., 2012; Tsai et al., 2012).

### Experimental Design and Statistical Analysis

Significant time and effort were initially spent to test several master transcription factors including NeuroD1 (ND1), Neurogenin2 (Ngn2; Supplementary Figure 1, Gangal et al., 2017; Jiang et al., 2019a,b), and Ascl1 to identify effective methods to efficiently carry out glia to neuron reprogramming. ND1 was eventually selected based on more than 2 years of *in vitro* and *in vivo* experiments and verification of combinations as well as individual applications of these factors. In cell culture experiments, at least three batches of cultures were tested per experiment, and the purity of astrocyte cultures was ensured by both GFAP staining and specific culture media/protocols. Multiple examinations were performed to independently replicate the virus infection efficacy and conversion efficiency, dose-response relationship, and cell phenotypes. In stroke animal experiments, the well-characterized focal ischemic stroke model with partial reperfusion represents common clinical cases (Wei et al., 2017; Zhong et al., 2020) and suitable for functional and behavioral tests. A specialized researcher with extensive rodent microsurgery training performed all ischemic surgery to ensure highly consistent ischemic insult in the sensorimotor cortex. Another experienced researcher performed all virus injection procedures to ensure consistent virus infections in the same brain region. The neuronal phenotype of reprogrammed cells was confirmed by at least two specific markers and the co-localization of cellular markers was examined by 3-D microscopy. We identified mature neurons not only rely on morphology and cell markers but also functional activity using electrophysiological recordings. In cell counting analysis, we followed stereology principles by separating analyzed by at least 100 μm to avoid double counting cells. In these experiments, sample sizes were six to eight per group and at least six sections were examined for each brain. In whole-cell recordings, over 20 cells per group were inspected; MEA recordings examined multiple brain slices from three to four brains per group. Functional and behavioral tests were performed using 6–14 animals per group, depending on the outcomes of Power analysis and our previous investigations. Data analysis was performed under blinded or double-blinded conditions.

All data were analyzed for normality using the D’Agostino and Pearson omnibus normality test (where *n* > 7) and Kolmogorov–Smirnov normality test (where *n* < 7). Unpaired Student’s *t*-test or Fisher’s test were used for pairwise comparisons. Comparisons of multiple groups were analyzed using one- or two-way ANOVA followed by *post hoc* Tukey’s test or other tests as indicated in figure legends. In these statistical reports, the variance between populations was tested using Bartlett’s test, F-factor, and *p-values* are provided. All results are expressed as Mean ± SEM Statistical comparisons were finished using Graph Pad Prism 6 (Graph Pad Software, Inc., San Diego, CA, USA). In general, *p* < 0.05 was considered significant for all comparisons; the exact *p-value* is reported for some important comparisons as a reference for readers.

## Results

**Figure 1:**
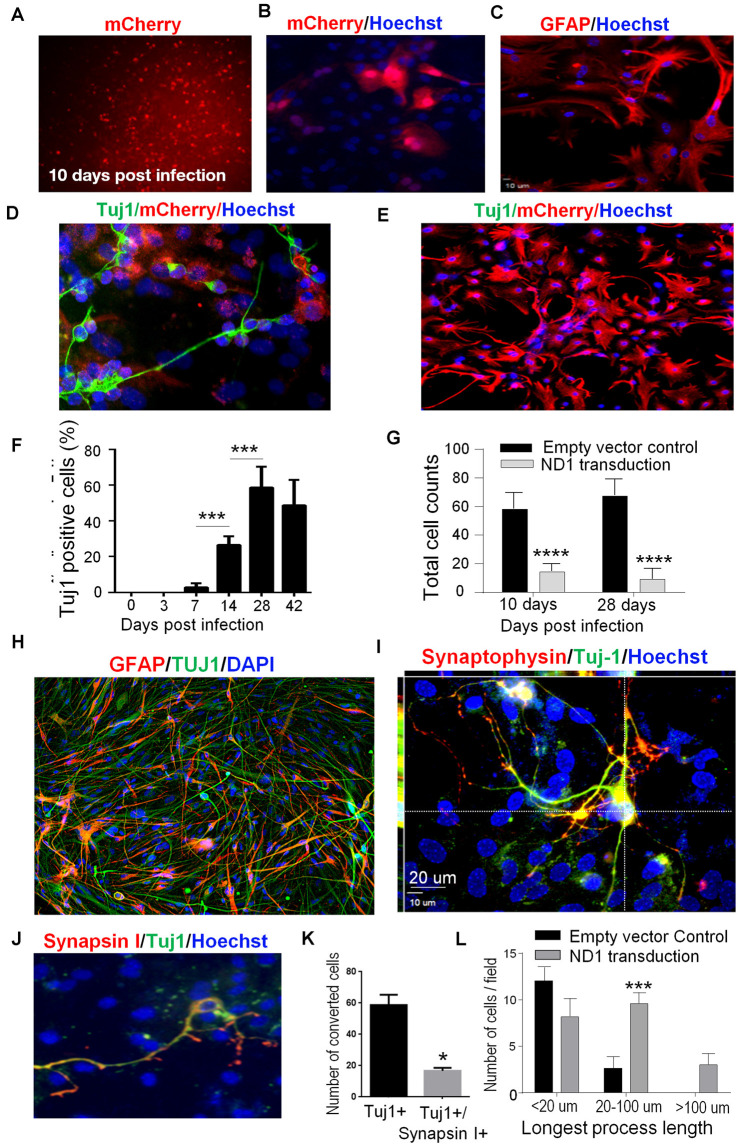
*In vitro* direct reprogramming of astrocytes to induced neurons. Mouse astrocyte cultures were transduced with NeuroD1 (ND1) or empty vector control and stained for different cell markers. **(A)** Ten days after lentiviral transduction, successfully infected cells were monitored over time by the expression of the fluorescent mCherry reporter (red), implicating GFAP-ND1-IRES-Ubi-mCherry expression in these astrocytes. **(B–D)** Enlarged images of infected cells 14 days after transduction. Infected cells expressed the reporter gene mCherry **(B)**, GFAP stain revealing an astrocyte-like morphology of astrocytes before reprogramming **(C)**. In image **(D)**, some cells develop the neuronal lineage marker Tuj1 (green) and adopted either a unipolar or bipolar morphology that is uncharacteristic of astrocytes. **(E)** A representative image of control culture with the empty vehicle virus showing the lack of Tuj-1 expression in infected cells (mCherry, red). There were also no alterations to the morphology of astrocytes. **(F)** Time course and reprogramming rate of the expression of the immature neuron marker Tuj1 in infected astrocytes up to 42 days after transduction. One-way ANOVA (*F*_(5,36)_ = 77.91) followed by Holm–Sidak’s multiple comparisons test. *N* = 3 independent cell culture batches, ***p < 0.001. **(G)** Quantified cell counts of total cell numbers 10 and 28 days after transduction with ND1 or vector control. The reduced cell number implied attenuated cell proliferation after the ND1 transduction and neuronal conversion. Two-way ANOVA (interaction: *F*_(1,10)_ = 2.377, *p* = 0.1542; time: *F*_(1,10)_ = 0.174, *p* = 0.6854; *treatment*: *F*_(1,10)_ = 358.9, ****p < 0.0001). **(H)** An immunostaining image shows coexpression of Tuj1 and GFAP in cultured astrocytes 6 weeks after transduction. **(I)** Confocal microscopy of an enlarged image from 6 weeks post-transduction, showing complex processes that are positive for Tuj-1 and synaptophysin colocalized with mCherry. Those cells under the culture condition did not show positivity to NeuN; however, they expressed the synaptic protein synaptophysin. **(J)** At 6 weeks after transduction, astrocytes undergoing direct reprogramming extended processes and developed nerve terminals positive to the pre-synaptic protein synapsin I (red). **(K)** The bar graph shows quantified data that at 6 weeks post-transduction, 58.7% of reprogrammed cells (mCherry-positive) expressed Tuj-1 and 17.4% of these cells also expressed synapsin 1. *n* = 3, paired *t*-test, *p < 0.05 vs. Tuj1 only cells. **(L)** Measurements of the length of extended processes from converted cells. There were drastically increased in the length of processes of cells received ND1 transduction. *N* = 3, two-way ANOVA (interaction: *F*_(3,9)_ = 8.427, ***p < 0.001).

Among single transcription factor-mediated direct reprogramming candidate genes, both ND1 and Ngn2 demonstrated an ability to reprogram reactive astrocytes but ND1 was selected based on both efficiency and efficacy (Supplementary Figure 1). A mCherry tagged ND1 lentivirus was generated under a mouse GFAP promoter and used for both *in vitro* and *in vivo* transduction. A viral titer of GFAP-ND1 was calculated to be approximately 4.8 × 10^7^ units/μl by using a 10^7^ dilution of virus that yielded ~92% infection in HEK293FT cells. Astrocyte cultures were infected at 7 days *in vitro* (DIV 7) and followed for up to 8 weeks post-infection to examine cell fate and efficiency of transduction and conversion. After lentiviral transduction, successfully infected cells could be monitored over time by the expression a mCherry reporter carried by the ND1 virus (Figures 1A–D). Infected cells began to adopt neuronal features around 2 weeks following transduction, including the extension of one or more long processes that are uncharacteristic of astrocytes (Figure 1C). While neuronal axonal processes are often elongated beyond the length of the cell body (>100 μm), astrocytes are characterized by multiple shorter and spread-out processes. Staining using the neuronal lineage market Tuj1 verified this morphological difference between mCherry-positive astrocytes and immature neuron-like cells by morphological examination (Figures 1D,H). On the other hand, astrocytes infected with the empty vector did not exhibit any of these alterations (Figure 1E). The observed cell lineage switch from ectoderm to endoderm is a time-dependent process. By 4 weeks, 58.6% of astrocytes were positive for the immature neuronal marker Tuj1 (Figure 1F). Consistent with the expected reduction in cell proliferation and survival rate of converted cells under culture conditions, the cell numbers in cultures that received ND1 transduction were drastically less than that in control cultures infected by the empty vector where astrocytes continued to proliferate (Figure 1G).

### *In vitro* Conversion of Astrocytes to Neuronal Cells

To confirm the maturity of astrocytes-converted iNeurons *in vitro*, we stained converted cells with the synaptic vesicle protein synaptophysin. As a marker for synapse formation, synaptophysin expression was detected in converted cells (Figures 1H,I). In the next experiment, we co-infected ND1 with a synapsin-GFP reporter to monitor cells. GFP expression emerged at 14 days post-transduction. In co-labeling experiments of Tuj1 and synapsin-1, around 20% of Tuj1-positive cells expressed the synaptic protein synapsin-1 (Figures 1J,K). During neuronal maturation, converted cells developed significantly longer processes (>20–100 μm) compared to cells infected by the control lentivirus, inspected at 6 weeks after transduction (Figure 1L).

**Figure 2:**
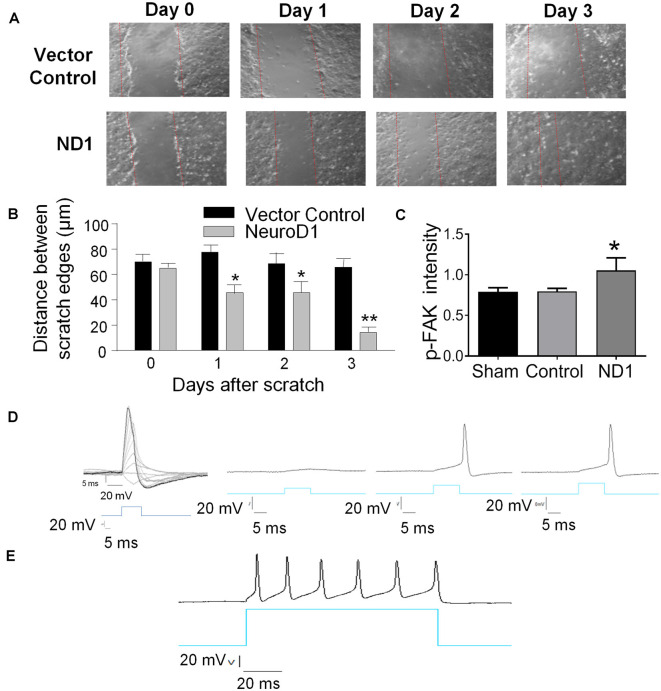
Reprogramming induced cellular changes and functional activities *in vitro*. The ND1-mediated astrocytes reprogramming was monitored in cultures and functionally characterized. **(A,B)** Two weeks after ND1 virus or empty control vector transduction, astrocyte cultures were challenged by the scratch test to measure the motility of reprogrammed cells. From day 1 after the scratch insult, the damaged area recovered significantly faster by migrating cells in cultures infected by ND1 compared to the vector control culture. *N* = 3 cell cultures. Two-way ANOVA (Interaction: *F*_(3,24)_ = 11.15, ***p < 0.0001; Time: *F*_(3,24)_ = 18.66, ***p < 0.0001; ND1: *F*_(1,8)_ = 53.87, ***p < 0.0001) followed by Holm–Sidak’s multiple comparisons test: *p < 0.05 and **p < 0.01 for ND1 vs. empty vector control. **(C)** Effect of the scratch test on the expression of migration factor focal adhesion kinase (FAK) in reprogrammed cells. In line with the increased motility 3 days after scratch, ND1-infected cells within 200 μm of the scratch expressed significantly higher immunofluorescence of phosphorylated FAK (p-FAK). *N* = 3 per group. One-way ANOVA, *F*_(2,6)_ = 7.327, **p = 0.0245 followed by Holm–Sidak’s multiple comparisons test: *p < 0.05 vs. control. **(D)** mCherry-positive converted cells were subjected to whole-cell recordings 28 days after infection. In the current-clamp mode, membrane depolarization induced by current injections evoked depolarization generated action potentials. A hyperpolarization was observed upon the decay phase of the spikes, which is typical for neurons and suggestive of functional potassium channels in these cells. *n* = 10. **(E)** A longer membrane depolarization pulse evoked the firing of a chain of action potentials that are characteristics of functional neurons.

Interestingly, reprogrammed cells also featured increased motility. In an *in vitro* scratch assay, reprogrammed cells moved into the scratched area much faster than vehicle control cells (Figure 2A). Three days later, the scratched area was significantly smaller compared to the control cultures (Figures 2A,B). Western blot analysis revealed that the phosphorylated focal adhesion kinase (p-FAK), a key mediator in cell adhesion and migration, was significantly increased (Figure 2C).

In electrophysiological examinations, the whole-cell recording was performed in mCherry-positive converted cells 28 days after ND1 transduction. In the current-clamp mode, gradually increased membrane depolarization triggered the firing of action potentials (Figure 2D). We patched a total of 10 mCherry positive cells with neuronal morphology; all cells exhibited sodium and potassium currents and action potentials upon membrane depolarization. Prolonged depolarization generated firing of repetitive action potentials, resembling functional activities of neuronal cells (Figure 2E).

### Focal Ischemic Stroke in Mice and mCherry-ND1 Lentivirus Injection

**Figure 3:**
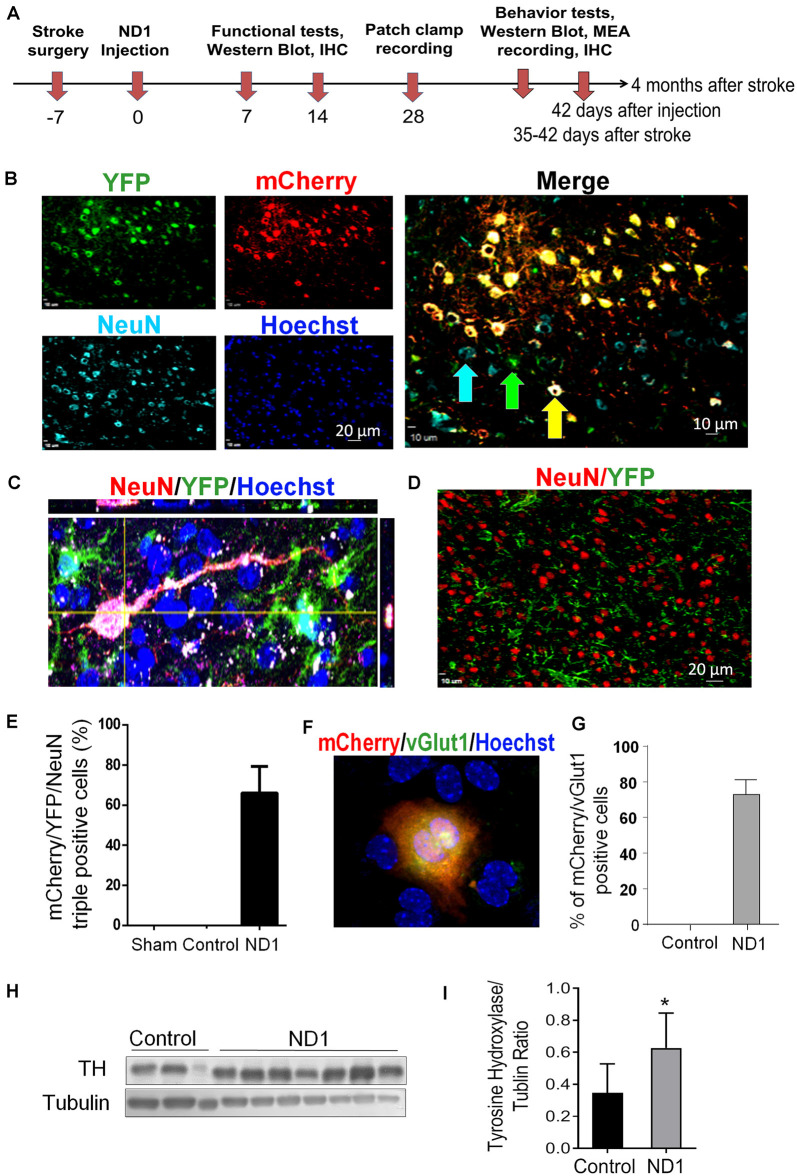
Conversion of astrocytes to mature neurons *in vivo*. Adult GFAP-Cre x Rosa-YFP mice were subjected to a focal ischemic insult to the right sensorimotor cortex and subjected to control and ND1 treatments. **(A)** Timeline of *in vivo* reprogramming experiments. Animals received sham, stroke control, or ND1 lentivirus injection to the peri-infarct region 7 days after stroke. empty control vector or ND1 lentivirus. Functional and psychological assessments were performed different days after stroke. Six weeks later, brain coronel sections were subjected to immunohistochemical staining with cell phenotype markers. **(B)** Representative images show cells expressing YFP (green, astrocytes), mCherry (red, ND1 infected cells), NeuN (light blue, mature neurons), and Hoechst 33342 (dark blue, nuclei of all cells). The enlarged merged image illustrates overlapped markers (yellow/orange), indicating astrocyte-converted iNeurons. **(C)** A high magnificent confocal 3-D image showing a converted iNeuron with overlaid markers mCherry (red, transfection marker), YFP (green, astrocytes origin), and NeuN (blue, mature neuronal marker). The converted cell adopted neuronal morphology of an extended axon. **(D)** Control experiment where the brain region was infected with empty control virus. Due to the lack of cell reprogramming and lineage change, the neuronal and astrocytes populations (NeuN of red color and YFP of green color) were distinctively located without co-localization. **(E)** Quantified data of the image analysis of mCherry/YFP/NeuN triple-positive cells. No such cell could be seen in sham and empty control vector cells. There were over 66.06% of mCherry/YFP/NeuN triple-positive cells among ND1-infected cells. *N* = 6 for sham or control, *n* = 8 for ND1 group. **(F,G)** At 3–4 weeks after ND1 transduction, the glutamatergic neuronal marker vGLUT (green) was detected and colocalized with mCherry (red; overlay color: yellow/orange) in the converted cells. The bar graph in **(F)** shows that more than 72.9% of converted cells expressed vGLUT. There was no vGLUT expression in empty vector control cells. *N* = 8 animals per group. **(H,I)** The dopaminergic neuronal marker tyrosine hydroxylase was detected *via* western blot in peri-infarct tissue from control and ND1 treated brains. The bar graph in **(H)** indicates a significant increase of the tyrosine hydroxylase (TH) level in the ND1 treated tissue compared to injection of the empty vector control. *N* = 3 independent brain samples for control and *n* = 7 for ND1. Unpaired *t*-test, *p < 0.05, *t* = 1.937, DF = 8; analysis of variance: *F*_(2,6)_ = 1.433, *p* = 0.9279.

Focal ischemic stroke of the right sensorimotor cortex was induced in adult GFAP-Cre × Rosa-YFP mice. Astrocytes in this mouse express the YFP reporter regardless of cell phenotype changes. This property is favorable for tracking astrocytes as they switch between inter-lineage phenotypes. After ischemic stroke, robust astrocyte accumulation took place in the peri-infarct region, and the ischemic core gradually forms a tissue cavity 2–3 weeks later (Kanekar et al., 2012; Pivonkova and Anderova, 2017). Proliferating astrocytes around the damaged tissue provided an abundant cell supply for reprogramming. Lentivirus containing mCherry-ND1 was injected into the peri-infarct region 7 days after ischemia, this time point was selected to avoid the acute phase when astrocytes are thought to be protective yet the virus transduction still catches hypertrophied astrocytes before the formation of a glial scar (Figure 3A).

### Reprogramming of Reactive Astrocytes and Neuronal Conversion After Ischemic Stroke

At 28 days after ND1 lentivirus injection to the peri-infarct region, approximately 10% of total astrocytes were YFP/mCherry double-positive cells. We identified cells labeled by YFP (identifying their astrocyte origin), mCherry (infected by ND1 lentivirus), and NeuN (mature neuron marker; Figure 3B). The combination of these three markers in single cells indicated successful viral-mediated reprogramming of endogenous astrocytes to neurons. Co-labeling of NeuN and transduction marker YFP in a single converted cell was also confirmed using confocal microscopy, showing a pyramidal neuronal phenotype with a single axon and dendritic arbors (Figure 3C). In contrast, cells infected by the empty control vector showed separated cell populations of neurons and infected astrocytes, illustrating high specificity of the viral promoter and no conversion in the absence of ectopic ND1 (Figure 3D). In cell counts, around 66% of infected cells became mCherry/YFP/NeuN triple-positive iNeurons (Figure 3E). Immunohistochemical examination revealed that most reprogrammed cells (~80%) expressed vesicular glutamate transporters (vGLUTs), which are the characteristics of glutamatergic neurons (Figures 3F,G). Western blotting of the peri-infarct tissue detected a significant increase in the expression of tyrosine hydroxylase (TH; Figures 3H,I), which is consistent with the notion that activation of the Wnt/β-catenin pathway and ND1 upregulates TH expression (Jiang et al., 2020). We did not observe the GABAergic neuronal marker glutamic acid decarboxylase 67 (GAD67; data are not shown). Measured at 14 days into the reprogramming process, none of the stemness markers Oct4 and Klf4 were detected in transduced (mCherry-positive) cells (Supplementary Figure 2), supporting that ND1-mediated glia-to-neuron conversion did not pass through an intermediate stem cell stage.

**Figure 4:**
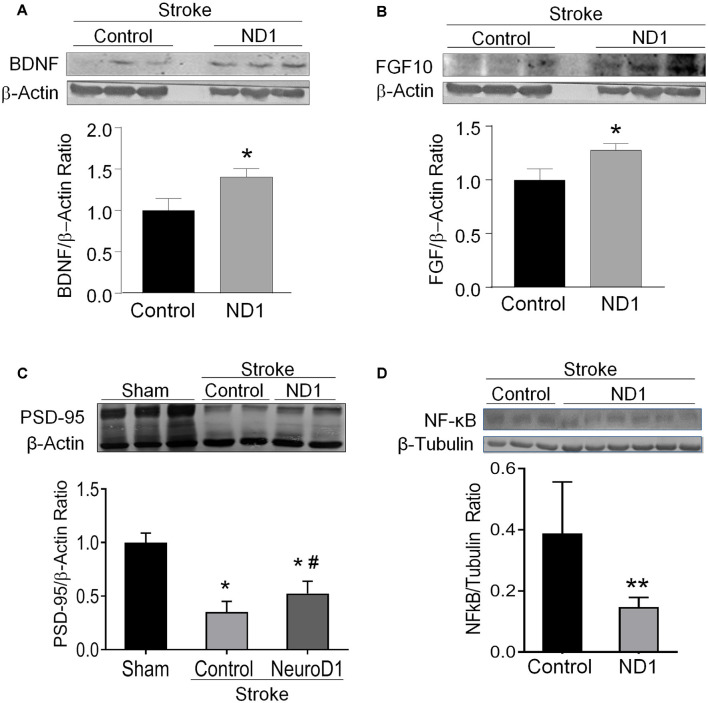
Increased expression of regenerative genes and brain protection after ND1 treatment. Western blotting measured several key growth factors and synaptic proteins in the peri-infarcted region following stoke in control and ND1-treated animals 6 weeks after stroke. **(A,B)** The expression of BDNF (unpaired two-tailed Student’s *t*-test: *n* = 8/group, *t* = 2.305, *p < 0.05) and FGF (unpaired two-tailed Student’s *t*-test: *n* = 8/group, *t* = 2.407, *p < 0.05) were significantly increased in the brain subjected to ND1 vs. vehicle control of stroke mice. Panels **(A,B)** were performed in the same Western blot experiments with the same load control. **(C)** The expression of postsynaptic protein PSD-95 decreased after stroke and ND1 treatment partially recovered this synaptic protein loss. Two-way ANOVA (interaction: *F*_(2,4)_ = 45.52, ***p = 0.0018 followed by Holm–Sidak’s Multiple Comparisons Test: *p < 0.05 vs. sham; ^#^*p* < 0.05 vs. empty vector control). **(D)** The ND1 treatment significantly reduced the NF-kB level in the post-stroke brain (unpaired two-tailed Student’s *t*-test: *n* = 3 for vector control and *n* = 6 for ND1 group, *t* = 3.613, **p < 0.01).

**Figure 5:**
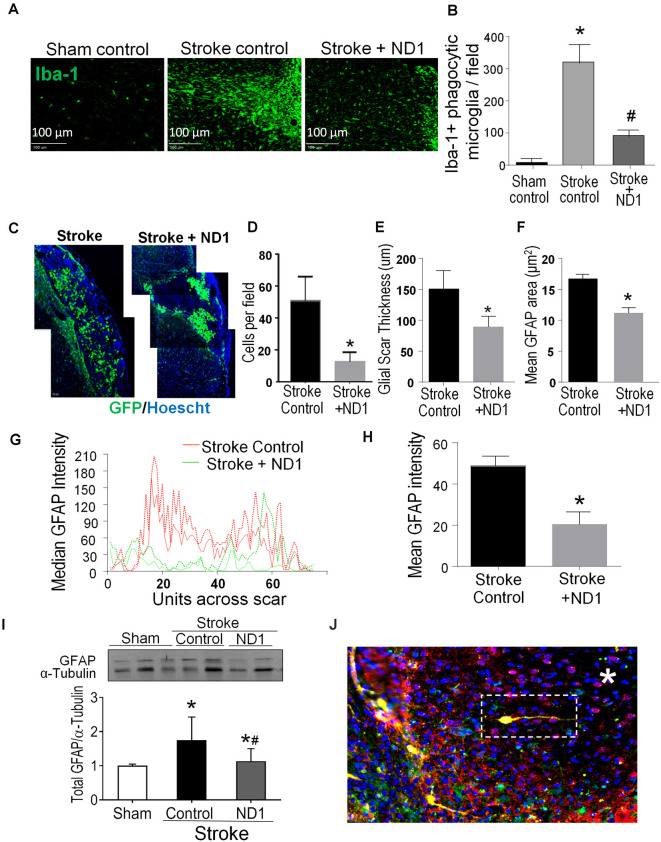
Direct reprogramming of astrocytes attenuated microglial activation and astrogliosis in the post-stroke brain. Inflammatory activities including microglia activation and astrogliosis were examined in the peri-infarct region. **(A,B)** Iba-1 expression was examined 6 weeks post ND1 infection. Iba-1 positive microglia are present in significantly greater numbers in the ipsilateral cortex compared to sham control or contralateral cortex. The iba-1 fluorescence of phagocytic microglia was markedly less in the ND1-treated cortex. **(B)** The number of microglia in the ischemic cortex was significantly increased after stroke; ND1 injection showed a large reduction in the number of microglia cells in the region. *N* = 5/group; one-way ANOVA (*F*_(2,12)_ = 118.4, *p* < 0.0001), *p < 0.05 vs. sham control, ^#^*p* < 0.05 vs. empty control vector. **(C–F)** Astrocyte accumulation or astrogliosis was evaluated in the peri-infarct region using immunohistochemical imaging 6 weeks after stroke after stroke. Images in C show accumulated astrocytes labeled by GFAP (green, arrows). The cell count **(D)**, the thickness of the gliosis **(E)**, and the mean GFAP area **(F)** were all significantly reduced. *N* = 6 per group paired *t*-test, *p < 0.05 vs. stroke controls. **(G,H)** Gliosis profile analysis by mean gray value across scar transection. The graph plots show median values of stroke control (red lines) and stroke plus ND1 (green lines). The bar graph in **(H)** quantified the measurement, demonstrating a significant reduction in GFAP positive astrogliosis after ND1 treatment. *N* = 3 paired *t*-test, *p < 0.05 vs. stroke controls. **(I)** Western blotting analysis of the GFAP level. The bar graph shows increased GFAP expression after stroke and the ND1 treatment attenuated this increase. *N* = 3, one-way ANOVA (*F*_(2,19)_ = 4.525, *p* = 0.0247), Holm–Sidak’s Multiple Comparisons Test; *p < 0.05 vs. sham, ^#^*p* < 0.05 vs. empty vector control. **(J)** An immunostaining image shows ND1-converted iNeurons (yellow) distributed inside the ischemic core (*). Cells were identified using mCherry (red), YFP (green), and NeuN (blue).

Neurotrophic/growth factors such as BDNF and FGF10 play critical roles in important processes such as cell survival, proliferation, and maturation, angiogenesis, neurogenesis, and wound healing, each of which plays an essential part in the regenerative process (Matkar et al., 2017; Spurlin and Nelson, 2017; Ghosh, 2019). Western blot assay in the peri-infarct region detected significantly higher levels of BNDF and FGF10 in ND1 transfected mice compared to animals who received empty control vector (Figures 4A,B). Consistently, the ND1 treatment significantly attenuated the stroke-induced reduction of the synaptic protein PSD-95 expression in the post-stroke cortex (Figure 4C). Meanwhile, the inflammatory marker protein NFκB expression and the number of phagocytic Iba-1-positive microglia were significantly lower in the ND1 treated brains than in stroke mice that received a control vector (Figures 4D, 5A,B and Supplementary Figure 3).

### Reprogramming of Reactive Astrocytes and Reduced Glial Scar After Ischemic Stroke

In stroke mice that received lentiviral ND1 administration, the number of YFP-positive cells in the peri-infarct area decreased compared to that in the stroke control cortex (Figures 5C,D). The relative size, thickness and mean fluorescent intensity of the GRAP-positive glial scar were also reduced (Figures 5D–H). 6 weeks after stroke, Western blot data revealed decreased GFAP levels in the peri-infarct region of ND1-treated stroke mice (Figure 5I). The reduced astrogliosis appeared to facilitate regenerative activities in the peri-infarct region. Some newly converted iNeurons (mCherry/YFP/NeuN triple-positive cells) were even observed inside of the core region where no neuronal cells would normally be expected to survive at this delayed post-stroke time point (Figure 5J).

### Astrocyte Reprogramming Improved Synaptic Transmission in the Peri-infarct Region

**Figure 6:**
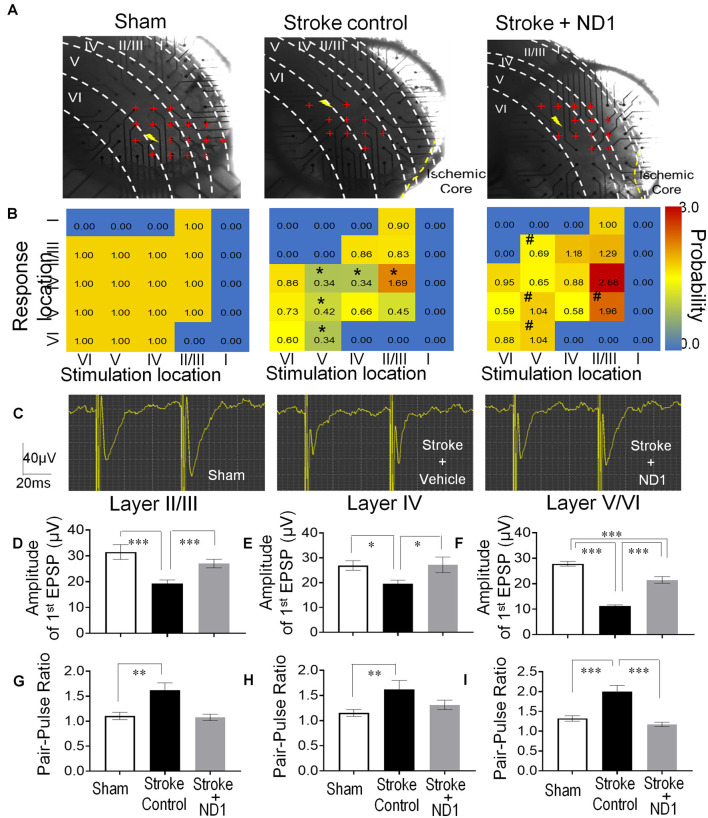
Functional repair of neuronal networks in the ischemic cortex. The Multi-Electrode Array (MEA) recording was performed to detect neuronal connections and functional activities in the sensorimotor cortex of the brain sliced 42 days after stroke. **(A)** Photos of brain slices and locations of 59 recording electrodes (red cross) crossing the six cortical layers (I, II/III, IV, V, and VI) near the ischemic core or the similar area of the sham recording. The shock sign indicates the location of the stimulation electrode and red cross indicates the electrodes or area where evoked EPSPs can be recorded. **(B)** Heat maps of evoked EPSPs showing the location and intensity of the response. Non-response areas are shown in blue color, and responsive areas of evoked EPSPs are shown in a yellow to red spectrum according to the response probability normalized to sham controls. After a stroke, the EPSP response likelihood was significantly reduced or even disappeared in II/III, IV, V, and VI cortical layers (*n* = 4–5 animals in each group, *p < 0.05, vehicle vs. sham controls, the Chi-square test). In recordings from slices subjected to ND1 transduction, there were more responsive areas (e.g., layer II/III) and the response probability of EPSPs was significantly greater than that in stroke controls (*n* = 4–5 animals in each group; ^#^*p* < 0.05 vs. stroke controls, the Chi-square test). **(C)** Representative recordings of the pair-pulse facilitation. **(D–F)** Quantified data summarized from the pulse facilitation recording in different cortical layers. The amplitude of the first EPSPs was significantly smaller compared to sham control and it was restored in ND1-treated slices [**(D)** *F*_(2,561)_ = 8.8, **(E)** *F*_(2,352)_ = 3.3, **(F)** *F*_(2,1173)_ = 89.7, *p < 0.05, ***p < 0.001, one-way ANOVA and Tukey’s *post-hoc*]. **(G–I)** The ratio of the second to first EPSPs was significantly larger after stroke, while the ND1 treatment returned the ratio to the normal level. *N* = 4–5 per group, **p < 0.05, ***p < 0.001 vs. sham or stroke controls [**(G)** *F*_(2,91)_ = 10.2, **(H)** *F*_(2,68)_ = 5.2, **(I)** *F*_(2,189)_ = 16.8, *p < 0.05, ***p < 0.001, one-way ANOVA and Tukey’s *post-hoc*].

The MultiElectrode array (MEA) system was used to record evoked extracellular post-synaptic potentials (EPSPs) in brain slices of sham control, stroke control of empty vector, and stroke with ND1 transduced mice (Figure 6A). In peri-infarct locations 6 weeks (42 days) after stroke, the responsive area covered by 59 recording electrodes and the amplitude of EPSPs evoked by a stimulation electrode were noticeably absent or reduced (Figure 6B). In the similar areas of slices from stroke mice that received ND1 transduction, normal or enhanced EPSPs were detected in multiple locations (Figure 6B). To further understand changes at the synaptic level, we examined the synaptic plasticity by paired-pulse facilitation (PPF). In PPF recordings, the second evoked EPSPs were large than the first one in all three experimental groups (Figure 6C). In fact, in cortical layers II/III, layer IV, and layer V we observed even greater facilitation in stroke control slices, which was partly due to a smaller size of the first EPSP in stroke control slices (Figures 6D–F). ND1 transduction prevented the smaller initial EPSPs and the abnormally enhanced synaptic facilitation (Figures 6D–I). Similar increased synaptic facilitation has been reported in human stroke patients (Kronenberg et al., 2014). Increased synaptic modification such as ischemia-increased long-term potentiation (LTP), has been called “pathological plasticity” (Frechou et al., 2019), which was ameliorated in ND1-treated slices (Figures 6G–I).

### Astrocyte Reprogramming Improves Functional Outcomes After Stroke

**Figure 7:**
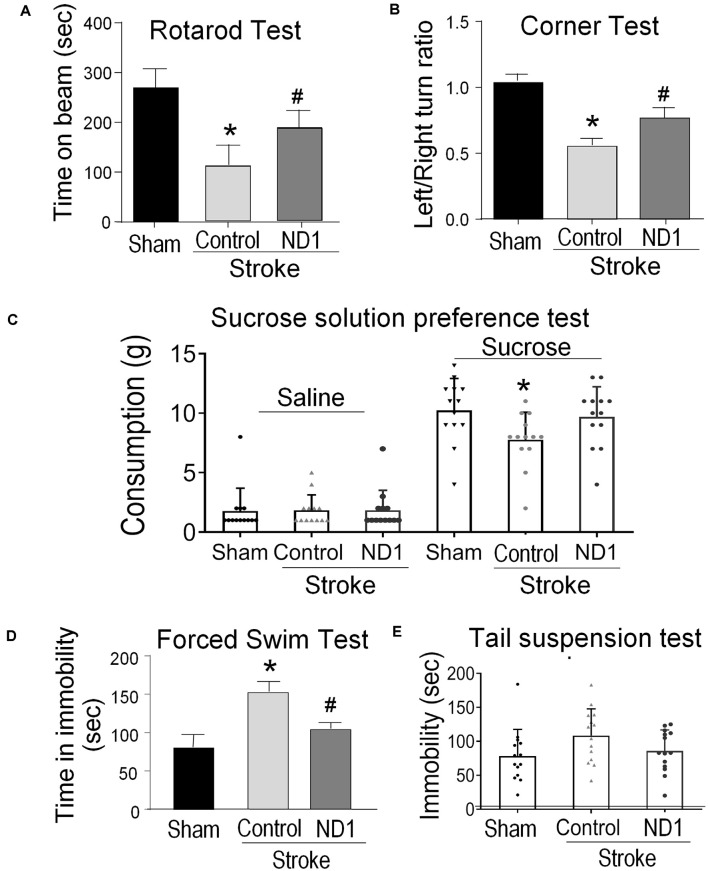
Direct conversion of astrocytes to neurons improved functional recovery after stroke. Functional and behavioral tests were performed different days after stroke to evaluate the therapeutic benefits of the direct conversion therapy using ND1 transduction. **(A)** The rotarod test was performed 21 days after stroke to measure the motor function of stroke animals. Stroke animals disabled a disability in balancing on the rotarod beam, their time spent on the beam was significantly shorter than sham control mice. ND1 conversion treatment noticeably improved the motor function of maintaining on the beam with a longer time. *N* = 6/group, two-way ANOVA (interaction: *F*_(3,44)_ = 29.9, ***p < 0.0001 followed by Bartlett’s test). Sidak’s multiple comparisons test: *p < 0.05 vs. Sham, ^#^*p* < 0.05 vs. vector control. **(B)** In the corner test, normal mice make equal left and right turns so the ratio of turns is close to 1.0. After a stroke, animals revealed a sensorimotor deficit of biased turn behavior due to the side of ischemic damage in the sensorimotor cortex. ND1 treatment showed a significant correction in this sensorimotor functional deficit. *N* = 6/group, two-way ANOVA (interaction: *F*_(2,15)_ = 19.91, ***p < 0.001 followed by Bartlett’s test). Sidak’s multiple comparisons test: *p < 0.05 vs. Sham, ^#^*p* < 0.05 vs. vector control. **(C)** Long-term psychological behaviors were tested 4 months after stroke. In the sucrose preference test, sham animals showed a marked increase in consuming the sucrose solution compared to saline, while stroke resulted in a significantly reduced interest in the sucrose solution. Stroke mice that received ND1 treatment maintained a similar interest in drinking the sucrose solution as sham mice. *N* = 12–14/group, one-way ANOVA (*F*_(2,36)_ 3.447, *p* = 0.0427), *p < 0.05 vs. Sham. **(D)** In the forced swim test, stroke mice exhibited significantly longer idle time in the water, indicative of a chronically developed post-stroke depression-like phenotype. This depression-like behavior was significantly corrected in stroke mice treated with ND1. *N* = 6/group, One-way ANOVA (*F*_(4,82)_ = 13.27, ****p < 0.0001) followed by Holm–Sidak’s multiple comparisons test: *p < 0.05 vs. before stroke, ^#^*p* < 0.05 vs. stroke control. **(E)** Tail suspension test of depression-like behavior in rodents. Stroke mice were observed to spend a longer time immobile compared to sham controls. ND1 treatment yielded a trend of reducing the immobility time. *N* = 14/group, one-way ANOVA (interaction: *F*_(2,39)_ = 2.53, ***p = 0.0926 followed by Bartlett’s test).

The focal ischemic stroke model results in well-defined motor and sensorimotor deficits detectable using the rotarod test and corner test (Grealish et al., 2016). Three weeks after stroke, the time that mice balanced on a rotating beam was significantly shorter compared to sham control mice (Figure 7A). Stroke mice that received the ND1 treatment, however, remained on the beam significantly longer than stroke mice received the empty control vector (Figure 7A). In the corner test, rodents normally make an equal left and right turn in their exploratory behavior. Stroke animals, however, show biased turns to one direction due to the unilateral injury to the right sensorimotor cortex (Figure 7B). Significant improvement in the turning behavior was seen with stroke mice received the ND1 treatment (Figure 7B). We and others have shown that stroke can induce chronically developed phenotypes of post-stroke depression-like behavior (Kronenberg et al., 2014; Frechou et al., 2019; Zhong et al., 2020). To further characterize long-term behavioral modifications, the sucrose solution preference test, forced swim test, and tail suspension test was performed 4 months after stroke. Stroke control mice exhibited obvious depressive-like behaviors in all these tests while no significant depressive-like changes were observed in mice that received ND1 treatment (Figures 7C–E).

## Discussion

The present investigation presents compelling evidence for the feasibility and effectiveness of utilizing reactive astrocytes as an endogenous cellular source for the generation of neuronal cells to repair damaged brain structures. Our *in vitro* experiments demonstrate the efficiency and the efficacy of the reprogramming process by ND1 as well as the neuronal activity of converted cells. Using an established sensorimotor ischemic stroke mouse model, we demonstrate that delivery of ND1 to reactive astrocytes in the peri-infarct region can be achieved in a temporally and spatially specific manner using a lentiviral vector. The ectopic expression of ND1 redirects reactive astrocytes into mature iNeurons, while reduced glial scar facilitates regenerative repair. MEA *ex vivo* recording revealed that ND1-induced reprogramming can restore disrupted cortical neuronal networks and correct pathological synaptic activity in the peri-infarct region. Finally, we observed improved short- and long-term functional and behavioral recovery with ND1 treatment, suggesting the potential to improve multiple outcomes as a result of this innovative therapy.

Glia to neuron reprogramming has recently been tested in basic and translational research (Grealish et al., 2016; Li and Chen, 2016). Early *in vitro* work demonstrated successful reprogramming of postnatal astrocytes and NG2 cells into functional neurons through forced expression of Pax6, Ngn2, or Ascl1 (Heins et al., 2002; Berninger et al., 2007; Heinrich et al., 2010). In the last few years, reactive astrocytes have been explored as a reprogramming pool in the adult mouse brain (Torper et al., 2013; Liu et al., 2020). Astrocytes are normally quiescent, and proliferation is not activated without injury (Ge et al., 2012; Tsai et al., 2012). In response to brain injury or neurodegeneration, astrocytes become reactive and exhibit changes in morphology, gene expression, and engage in rapid proliferation (Sofroniew and Vinters, 2010; Robel et al., 2011). Reactivity of astrocytes to brain injury provides an advantageous step in the expression of transcription factors using the GFAP promoter to drive the expression of ectopic transcriptions factors and reprogramming of these cells.

Reactive astrocytes in the peri-infarct region encase the injury site and play a biphasic role during acute and chronic phases of stroke (Sofroniew and Vinters, 2010; Robel et al., 2011). At the early phase after ischemic stroke, accumulating astrocytes limit the expansion of tissue damage. At later phases after stroke, the formation of a physical and chemical barrier between the ischemic core and surrounding regions is inhibitory for regeneration. The glial scar comprised primarily of astrocytes secretes inflammatory and growth-inhibitory factors, i.e., TNF-α, Interleukins, and proteoglycan proteins that inhibit neurite outgrowth (Karimi-Abdolrezaee and Billakanti, 2012; Huang et al., 2014). This physical and chemical barrier during the later stage of stroke becomes a major hurdle for neuronal regeneration and tissue repair. In contrast to this theory, a recent study in a spinal cord injury model suggested that reactive astrocytes play an essential role in neuronal repair (Anderson et al., 2018). We presume that the role and process of astrogliosis may differ in the spinal cord and the brain. Nevertheless, the goal of conversion therapy is not to eliminate astrocytes in the local region. The strategy of converting only a portion of astrocytes aims to the remaining cellular homeostasis and supporting role of astrocytes while facilitating neuronal regeneration in the local microenvironment.

Recent evidence suggests that low-level neurogenesis can occur outside the two canonical niches in SVZ and SGZ. Neuroblasts have been observed in the striatum, cortex, and amygdala of rodents, rabbits, guinea pigs, and primates including humans (Ernst et al., 2014; Luzzati et al., 2014; Feliciano et al., 2015). Local generation of new neurons has also been reported in the cerebral cortex, where layer I progenitors traced by retrovirus-mediated labeling were shown to produce neurons (Ohira et al., 2010). These events imply that endogenous neurogenic activities do take place in different brain regions for tissue homeostasis and neuroplasticity. It is also likely that these same processes are enhanced after brain injuries such as stroke (Magnusson et al., 2014). This had led some to suggest that astrocytes already represent quiescent neuronal progenitors that can become activated after injury. Based on this view, the reprogramming strategy provides a method of enhancing this endogenous mechanism to further enforce the neurogenic competence of reactive astrocytes proximal to the site of injury.

Reactive astrocytes in the peri-infarct region may arise from remote areas including the SVZ (Niu et al., 2013). The present investigation was not intended to differentiate between local astrocytes and those that had migrated to the site. A major challenge and necessary task are to distinguish converted iNeurons from preexisting neurons and their relative contribution to tissue repair. Proteins that are specific to astrocytes are not typically expressed in mature neurons. Thus, an astrocyte-specific reporter cannot be used to trace converted cells. To this end, we employed a Cre-lox system to permanently switch on the expression of a fluorescent protein so that astrocytes could be traced throughout the reprogramming process regardless of cell phenotype transformation. Our Cre-lox-dependent YFP expression is “locked-in” before the injury and before the upregulation of GFAP expression in astrocytes, enabling us to determine the astrocyte origin of converted neurons by YFP expression.

Alterations in the epigenetically regulated enzymes, variations in DNA methylation, histone modifications, and chromatin accessibility have been shown to occur in glial cells upon spontaneous neurogenic activation or reprogramming (Endo et al., 2011; Holtzman and Gersbach, 2018). Current reprogramming strategies can generate both excitatory glutamatergic and inhibitory GABAergic neurons in the adult brain and spinal cord (Torper et al., 2013; Guo et al., 2014; Liu et al., 2015; Gascon et al., 2016). Consistent with these observations, we observed that ND1 primarily reprogrammed astrocytes into glutamatergic neurons, which are the most populous in the cerebral cortex. Since iNeurons are derived from a patient’s own brain cells, they are compatible with the host and avoid possible rejection by the immune system or triggering inflammatory reactions. As shown in this investigation, ND1 mediated conversion did not trigger augmented inflammatory reactions; rather, it suppressed microglial activation and NFκB expression.

Aside from ND1 mediated direct reprogramming, alternative methods to increase neuronal production have been explored. For example, the removal of the p53-p21 pathway has also yielded significant promotion of direct reprogramming (Wang et al., 2016). Similarly, depletion of the RNA-binding protein PTBP1 also converted human astrocytes to functional neurons in a Parkinson disease model (Gao et al., 2017). Certain neurotrophic factors have been shown to enhance the rate of direct conversion into neurons (Zhang et al., 2015). Supplemental treatments or co-expression of factors such as BDNF should also promote the maturation and survival of induced neurons (Zhang et al., 2015). Pathways involved in oxidative stress may act as key metabolic checkpoints during the glia-to-neuron conversion (Qian et al., 2020). Treatments with small molecules, such as VPA, calcitriol, or α-tocotrienol, may be tested since they also enhance neuronal survival and maturation, in addition to increased glia-to-neuron conversion (Su et al., 2014; Niu et al., 2015; Gascon et al., 2016). Cocktails of small molecules have been reported for the neuronal conversion of cultured human astrocytes (Zhang et al., 2015; Gao et al., 2017). Lastly, safety and efficiency are of paramount importance when these techniques are considered for clinical applications. Our study provides proof-of-principle data using a temporally and spatially specific lentivirus approach. Future research is required to explore more virus-mediated reprogramming therapies such as the non-integrating AAV vectors and non-virus approaches to prevent potential mutagenesis of genes critical for normal cell function or tumorigenesis. A balance must be maintained between the production of iNeurons with a supportive microenvironment to ensure safe and effective repairs at different time points after stroke. A better understanding of the molecular mechanisms underlying neuronal reprogramming may allow further flexibility in reprogrammed cell fate for specific brain disorders. Appropriate neuronal reprogramming schedules need to be explored for various applications in different injury models. Delayed reprogramming experiments are also needed to elucidate the therapeutic time window of this therapy.

## Conclusions

The direct reprogramming of glial cells to neurons provides an unprecedented opportunity to repair damaged brain structures by a novel form of neurogenesis. This strategy utilizes an enriched endogenous cellular pool following injury, allowing on-site repair using autologous cells that are already integrated into the host tissue extracellular matrix. This approach also synergistically alters the pathology of stroke by ameliorating the inhibitory glial scar and associated inflammatory cascades. While this potential therapy is promising, it is also under debate due to previous inconclusive results. Data from this comprehensive investigation support that the glia-neuron conversion strategy is an effective and functionally beneficial therapy for ischemic stroke.

## Data Availability Statement

The original contributions presented in the study are included in the article/Supplementary Material, further inquiries can be directed to the corresponding author/s.

## Ethics Statement

The animal study was reviewed and approved by Emory University IACUC.

## Author Contributions

SY and LW: conceptualization, resources, supervision, and funding acquisition. MJ, ZW, WZ, WC, XG, AW, MM, and KB: methodology and investigation. ZW and MJ: validation. MJ, ZW, and WZ: formal analysis. SY: data curation and project administration. SY and MJ: writing—original draft preparation. SY, MJ, MM, and KB: writing—review and editing. SY, MJ, AW, and LW: visualization. All authors have read and agreed to the published version of the manuscript. All authors contributed to the article and approved the submitted version.

## Conflict of Interest

The authors declare that the research was conducted in the absence of any commercial or financial relationships that could be construed as a potential conflict of interest.
