# Supplementary material for: Conversion of Reactive Astrocytes to Induced Neurons Enhances Neuronal Repair and Functional Recovery After Ischemic Stroke
Source: Front Aging Neurosci. 2021 Mar 26;13:612856. doi: 10.3389/fnagi.2021.612856 (PMC8032905; doi:10.3389/fnagi.2021.612856)
Supplement: Supplementary file 1 [file Table_1.DOCX]

**Supplemental figure legends**

***Suppl. Figure 1 Direct reprogramming by single transcription factor transfection using Ngn2 and NeuroD1.***

**A**. Lentiviral Construct motif for NeuroD1 and Ngn2. **B.** Experimental timeline of *in vitro* lentiviral transfection. 293FT cells in 35mm dish were infected with 0.2 μl virus and fixed 4 days later. **C.** EIMW-Ngn2-myc transfected 293FT cells express mCherry and myc after 4 days in vitro. **D.** Direct reprogramming *in vitro* 2 weeks after virus infection of mouse astrocyte cultures. Both NeuroD1 and Ngn2-myc lentivirus were able to reprogram mouse astrocytes into neuronal cells after 14 days in vitro. NeuroD1 (ND1) was selected in the investigation because of its higher efficacy.

***Suppl. Figure 2. ND1 mediated conversion of astrocytes did not pass through a stem-like stage***

To determine if ND1 transduced cells pass through a pluripotent intermediate, we assessed the expression of stemness markers 14 days after infection in the mouse cortex at the site of the injection. In mCherry positive ND1 transduced cells **(A)**, we did not detect stemness markers Oct4 **(B)**, Sox2 **(C)**, and Klf4 **(D)** in the mouse brain, supporting the idea that ND1 expression directly converted astrocytes into induced neurons without passing through an intermediate stem cell stage.

***Suppl. Figure 3. Microglia cells before and after stroke and ND1 treatment***

Immunostaining of Iba-1 positive microglia in the sham control and stroke brain. **A**. Images of Iba-1 labeled microglia exhibited different morphologies. The white arrow points to ramified microglia with long branches that a commonly seen in the adult normal brain. The red arrow head points to activated phagocytic microglia with thickening and retraction of branches associated with neuroinflammation. **B** and **C**. The total Iba-1 positive microglia were similarly increased in the peri-infarct region of stroke animals with and without ND1 treatment (B). ND1-treated brain, however, showed significantly fewer phagocytic microglia likely due to attenuated inflammatory reactions (C).
