# Supplementary figures and images for: Conversion of Reactive Astrocytes to Induced Neurons Enhances Neuronal Repair and Functional Recovery After Ischemic Stroke
Source: Front Aging Neurosci. 2021 Mar 26;13:612856. doi: 10.3389/fnagi.2021.612856 (PMC8032905; doi:10.3389/fnagi.2021.612856)

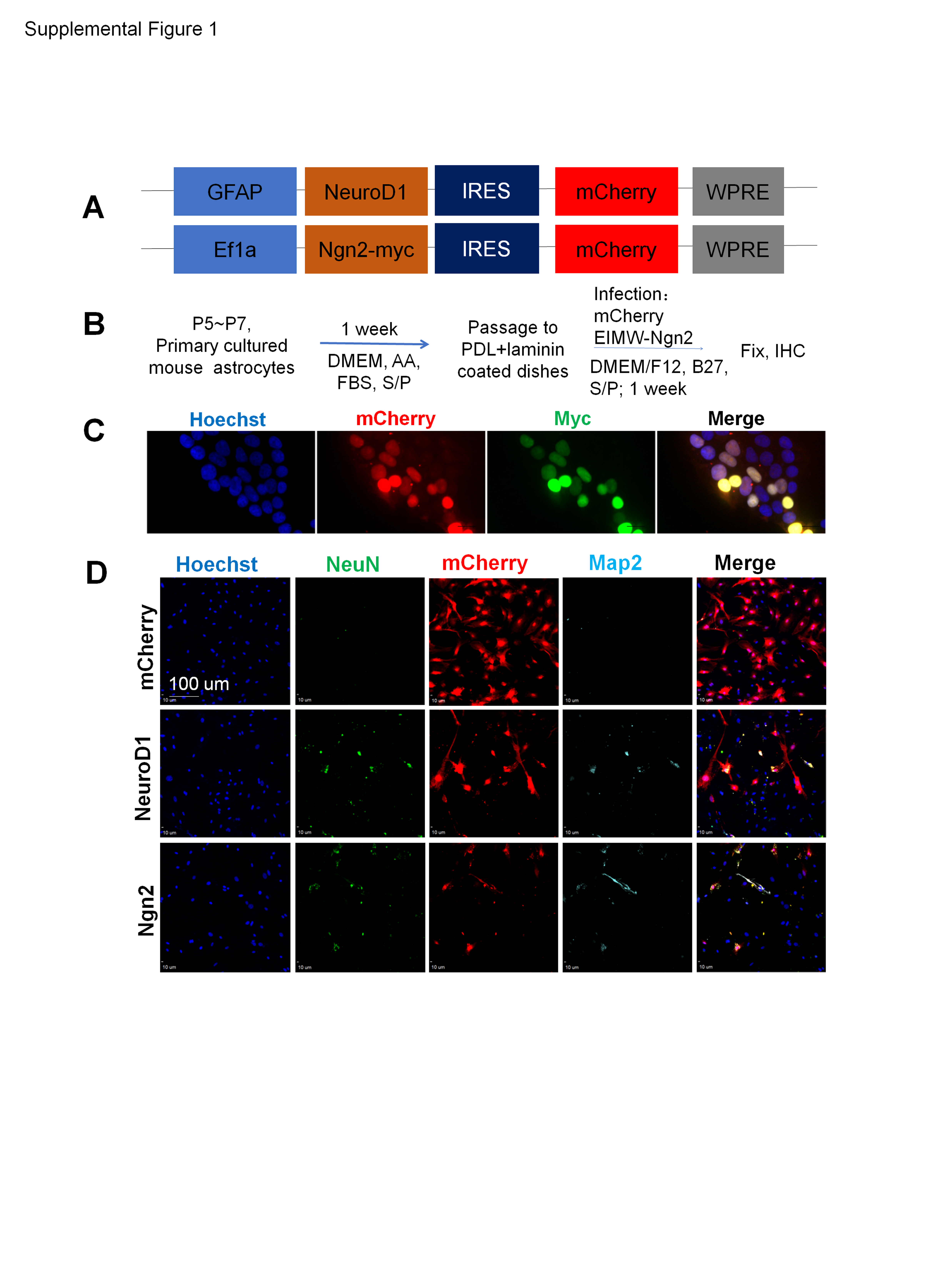

Supplement: Supplementary file 2 [file Image_1.TIF]

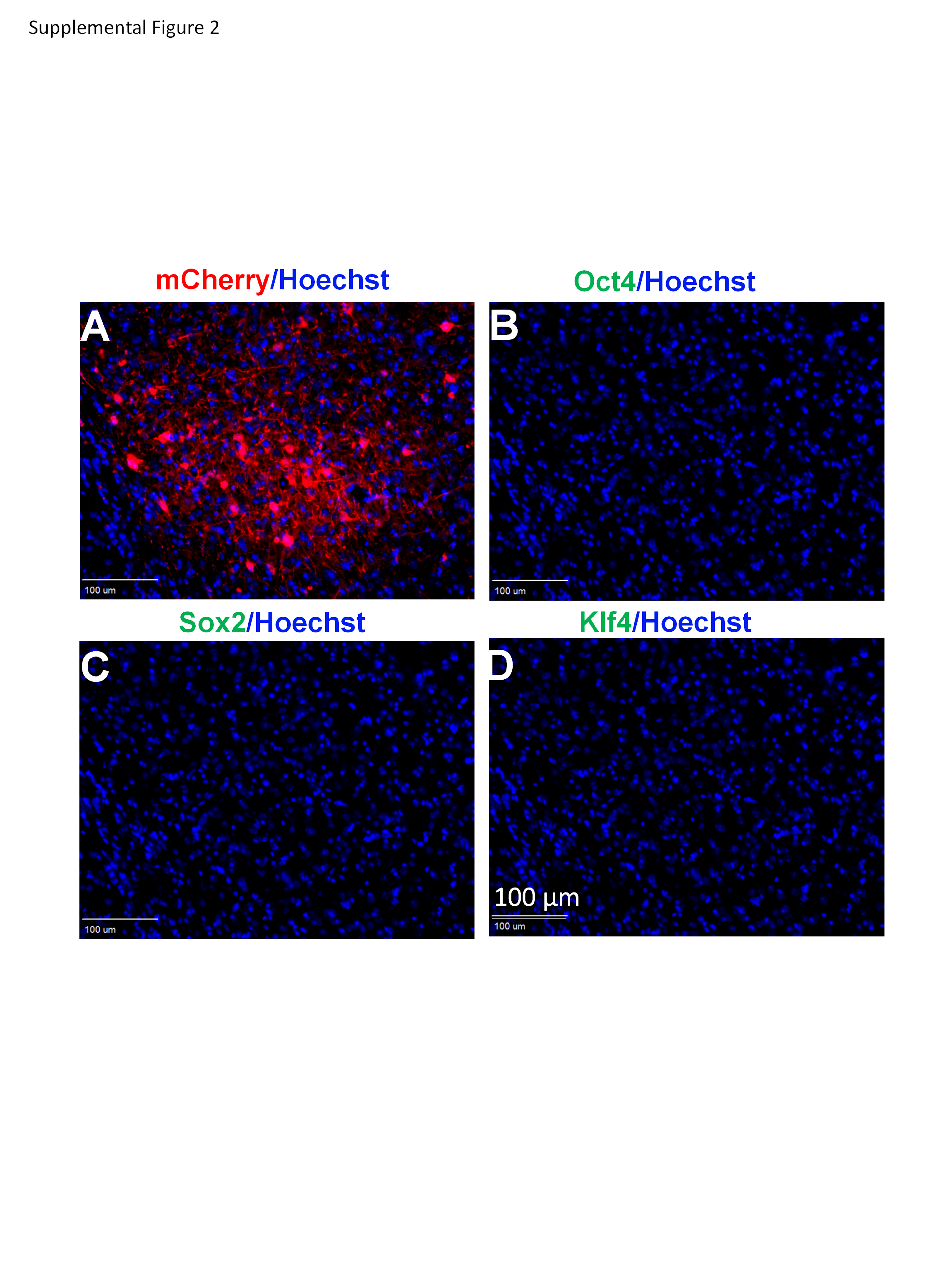

Supplement: Supplementary file 3 [file Image_2.TIF]

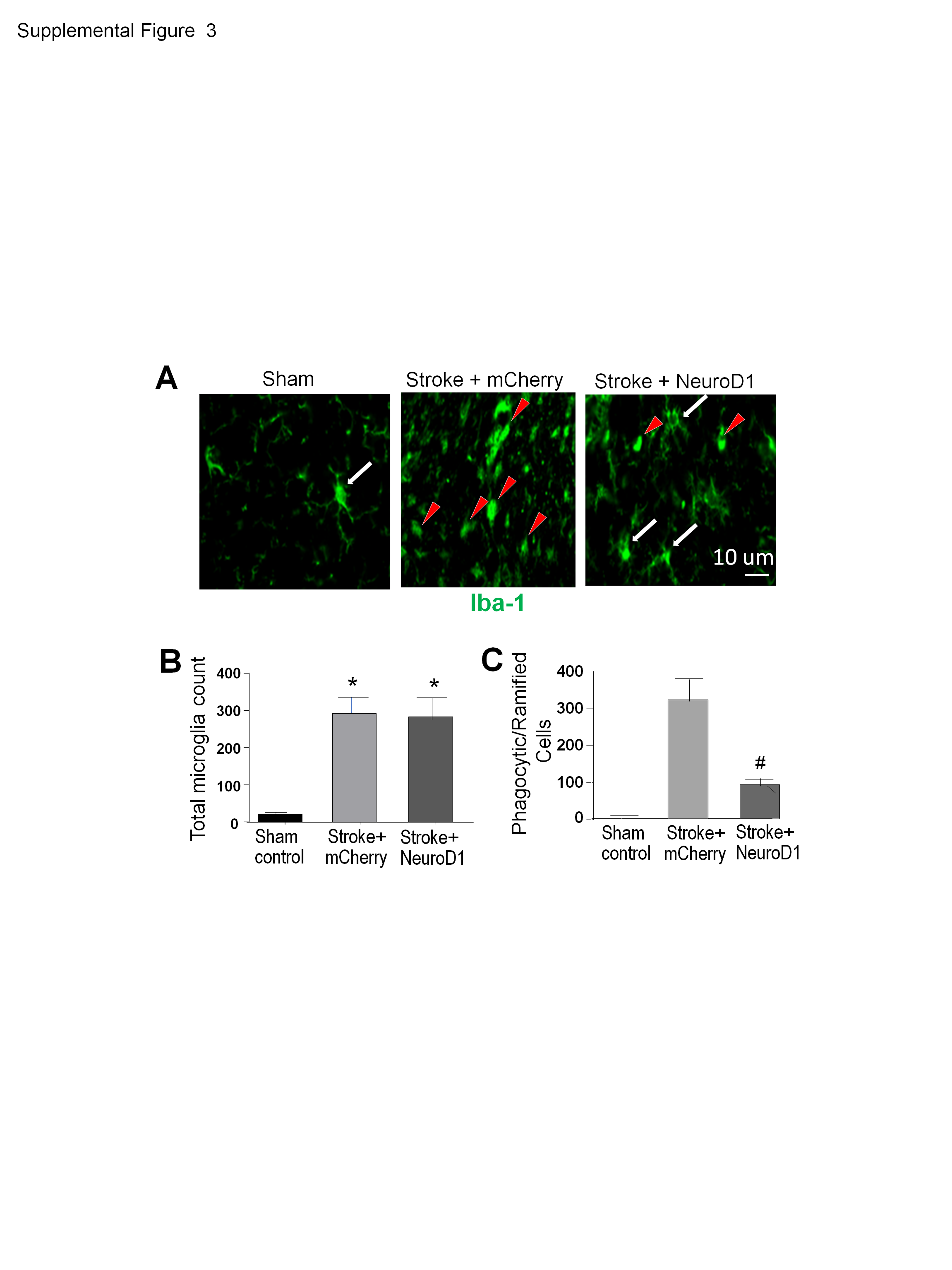

Supplement: Supplementary file 4 [file Image_3.TIF]
